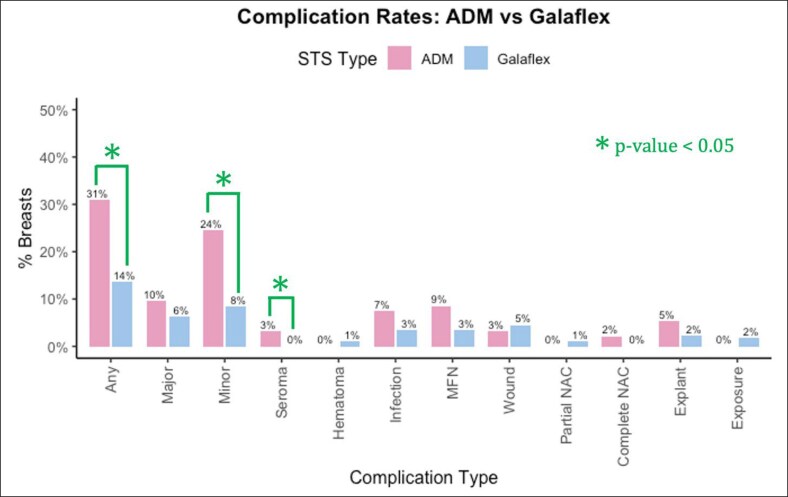# A Comparative Analysis of Poly-4-hydroxybutyrate (P4HB) and AlloDerm Soft Tissue Support in Direct-to-Implant Breast Reconstruction

**DOI:** 10.1093/asjof/ojag138.018

**Published:** 2026-07-24

**Authors:** Lauren E Romanowski, Kshipra Hemal, Thomas J Sorenson, Carter J Boyd, Chris Amro, Sofia Perez Otero, Raeesa Kabir, Oriana Cohen, Mihye Choi, Nolan S Karp

**Affiliations:** Hansjörg Wyss Department of Plastic Surgery, NYU Langone, New York, New York; Hansjörg Wyss Department of Plastic Surgery, NYU Langone, New York, New York; Hansjörg Wyss Department of Plastic Surgery, NYU Langone, New York, New York; Hansjörg Wyss Department of Plastic Surgery, NYU Langone, New York, New York; Hansjörg Wyss Department of Plastic Surgery, NYU Langone, New York, New York; Baylor College of Medicine, Division of Plastic Surgery, Houston, TX; University of Minnesota School of Medicine, Minneapolis, MN; Hansjörg Wyss Department of Plastic Surgery, NYU Langone, New York, New York; Hansjörg Wyss Department of Plastic Surgery, NYU Langone, New York, New York; Hansjörg Wyss Department of Plastic Surgery, NYU Langone, New York, New York

## Abstract

**Purpose:**

Implant-based breast reconstruction (IBBR) remains the predominant post-mastectomy reconstructive modality, representing 79% of all breast reconstructions in 2024.^1^ The use of softtissue support (STS) has further advanced pre-pectoral IBBR by providing additional pocket stability.^2,3^ Traditionally, acellular dermal matrices (ADMs) comprised the predominant form of STS, used in 51% of 2024 reconstructive breast procedures.^1^ However, ADMs are not without limitations, including added costs and potentially higher rates of complications, facilitating investigations into other biologic and synthetic STS options.^4,5^

Poly-4-hydroxybutyrate (P4HB) is a synthetic, fully absorbable mesh with established use within aesthetic breast surgery.^6^ Given its favorable long-term performance, P4HB may offer advantages in pre-pectoral direct-to-impact (DTI) IBBR by reducing dead space, stabilizing the implant pocket, and minimizing micro-shift. Subsequently, this study compares clinical outcomes of P4HB with ADMs in pre-pectoral DTI IBBR.

**Methods:**

A retrospective review of all consecutive patients who underwent mastectomy followed by pre-pectoral DTI reconstruction between July 2017 and December 2024 was performed. Patients were grouped by STS type and analyzed by patient and by breast; bilateral cases received the same STS in each breast.

Primary outcomes included rates of overall complications, classified as either major (e.g., necessitating readmission or reoperation) or minor (e.g., managed with outpatient care, antibiotics, or debridement). STS type was evaluated as a predictor using multivariable regression and propensity-score matching.

**Results:**

A total of 153 patients (271 breasts) met inclusion criteria. P4HB was used in 102 (67%) breasts, and ADM was used in 51 (33%). Mean follow-up was 13 months and did not differ by STS type. Patient demographics were similar between groups; on average, patients were 50 years old, non-smokers (100%), non-diabetics (94%), and had a median body mass index (BMI) of 25 kg/m2 . BMI was lower in the P4HB cohort (24 vs. 27 kg/m2 , p<0.05).

Thirty-four (22%) patients neoadjuvant received chemotherapy and 15 (6%) breasts underwent neoadjuvant radiation, with no significant differences in rates by STS type. Most mastectomies were nipple-sparing (n=176, 65%), and P4HB was preferentially used following NSM (71% vs. 29%, p<0.05). p<0.05). Median mastectomy weight was 514 grams, which was lower in the P4HB cohort (444 vs. 653 grams, p<0.05).

Overall complications occurred in a total of 53 (19%) breasts, with major complications in 20 (7%) breasts and minor complications in 38 (14%) breasts. The rate of overall and minor complications was significantly lower in P4HB breasts (14% vs. 31% and 9% vs. 25%, p<0.05). Seromas occurred in 3 (3%) breasts, all of which used ADM (3% vs. 0%, p<0.05).

After controlling for confounders in multivariable analysis and propensity-score matched analysis, STS type was not found to be a significant predictor of overall or minor complications. Conversely, higher BMI was associated with higher odds of overall complications (OR 1.1, p<0.05). A previous history of radiation was associated with higher odds of minor complications (OR 4.5, p<0.05), which held true on propensity score matching (adjusted OR 8.7, p<0.05).

**Conclusions:**

P4HB soft-tissue support was associated with fewer overall complications, fewer minor events, and no seromas on unadjusted analysis. Although these differences were not sustained after risk adjustment, P4HB performed equivalently to ADM despite being used in smallermastectomy-weight and nipple-sparing cases. These findings support P4HB as a safe, effective, and aesthetically favorable synthetic alternative to ADM in pre-pectoral DTI reconstruction. Further prospective studies are warranted to refine patient selection and evaluate long-term reconstructive and aesthetic outcomes with P4HB.

**Figure:**